# Rapid tumor regression in an Asian lung cancer patient following personalized neo-epitope peptide vaccination

**DOI:** 10.1080/2162402X.2016.1238539

**Published:** 2016-10-07

**Authors:** Fenge Li, Caixia Chen, Tao Ju, Junqin Gao, Jun Yan, Peng Wang, Qiang Xu, Patrick Hwu, Xueming Du, Gregory Lizée

**Affiliations:** a Department of Gynecology, Tianjin First Center Hospital, Tianjin, China; b Tianjin HengJia Biotechnology Development Co., Ltd, Tianjin, China; c Department of Oncology, Tianjin Beichen Hospital, Tianjin, China; d Pathology Department, Tianjin Beichen Hospital, Tianjin, China; e Pathology Department, Tianjin First Center Hospital, Tianjin, China; f Regenerative Medicine Center, Third Central Hospital, Tianjin, China; g GenomiCare Biotechnology Co. Ltd., Shanghai, China; h Department of Melanoma Medical Oncology, University of Texas M.D. Anderson Cancer Center, Houston, TX, USA; i Department of Immunology, University of Texas MD Anderson Cancer Center, Houston, TX, USA

**Keywords:** Cancer vaccine, cytotoxic T cells, EGFR L858R mutation, epidermal growth factor receptor, lung cancer, neo-epitope, personalized immunotherapy

## Abstract

Personalized immunotherapy targeting tumor-specific mutations represents a highly promising approach to cancer treatment. Here, we describe an Asian lung squamous cell carcinoma patient demonstrating frank disease progression following chemotherapy and EGFR inhibitor treatment. Based on tumor mutational profiling and HLA typing, a saline-based multi-epitope peptide vaccine was designed and administered along with topical imiquimod as an adjuvant. Weekly neo-epitope peptide vaccination was followed by a rapid and dramatic regression of multiple lung tumor nodules, while a much larger liver metastasis remained refractory to treatment. Peripheral blood immune monitoring showed that specific cytotoxic T lymphocytes (CTLs) were induced primarily against peptide targets encompassing the widely shared EGFR L858R mutation, particularly one restricted to HLA-A*3101. Immunological targeting of this driver mutation may be of particular benefit to Asian lung cancer patients due to its relatively high prevalence within this patient population.

## Introduction

The well-described carcinogenic impact of tobacco smoke makes lung cancer one of the most highly mutated of all cancers.^1-2^ Recent large-scale clinical studies have shown that response rates of cancer patients to checkpoint blockade-based immunotherapy are well correlated with mutational burden, predicting that lung cancers may be particularly responsive to immunotherapeutic interventions.^3-5^ Although checkpoint-based approaches provide non-specific immune activation, this correlation suggests that the antitumor immune responses are mediated largely through the activation of T lymphocytes recognizing mutated peptides presented by HLA molecules at the tumor cell surface.^6-8^ Although identification of mutated peptide epitope targets in individual patients remains a daunting technical challenge, recent advances in next generation genetic sequencing has provided a strong foundation on which to build these efforts.^9-10^ Overcoming this hurdle holds the promise of a more personalized and focused approach to activating antitumor immunity, without the off-target autoimmune side effects frequently observed with checkpoint blockade.^11-12^ Recent vaccination approaches employing mutated peptides to stimulate antitumor immunity have shown success in generating specific cytotoxic T lymphocyte (CTL) responses in human melanoma patients, and similar approaches in murine models have resulted in substantial tumor regressions.^13-14^ These highly encouraging studies have led to the initiation of several mutated peptide vaccine trials aiming to test whether such clinical benefits are translatable to human cancer patients.^15-16^

### Case report

**Figure 1.**
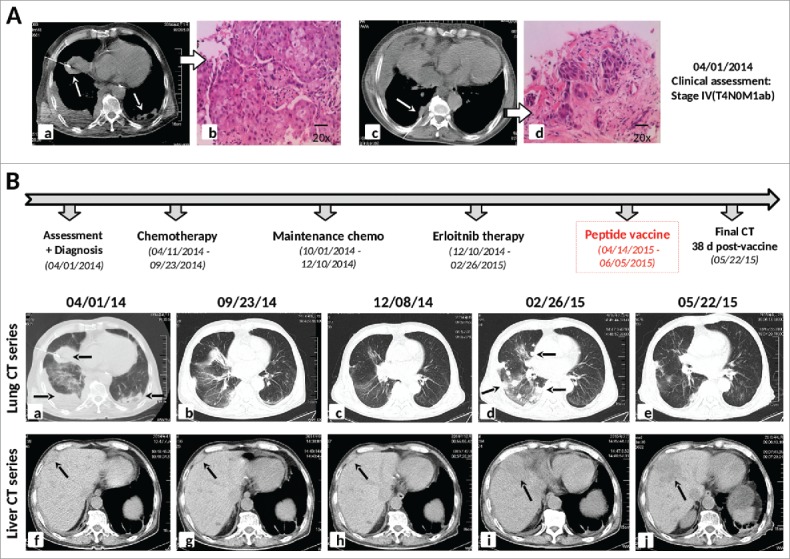
Diagnostic assessment, treatment, and clinical monitoring of lung cancer patient. (A) Pre-treatment assessment of Stage IV lung squamous cell carcinoma (T4N0M1ab), as determined by chest CT scans *(a,c)* and pathological examination of H&E stained needle biopsies *(b,d)*. (B) Timeline indicating treatment dates of frontline and maintenance cycles of Vinorelbine chemotherapy, erlotinib treatment, and personalized peptide vaccination. The corresponding chronological series of chest and abdominal CT scans are shown, from initial patient assessment *(a,f)*, during and following chemotherapy *(b,c,g,h)*, following erlotinib treatment *(d,i)*, and 38 d following the initiation of personalized peptide vaccination *(e,j)*.

A previously healthy 85-y-old man of Asian descent was admitted to the Oncology Department of Beichen Hospital (Tianjin, China) in March 2014 due to chest pain, cough, fever, and difficulty breathing.  Blood tests were normal, showing no evidence of potential infection. Computed tomography (CT) chest scans revealed an irregular mass (3.5 × 4.2 × 6.9 cm) in the hilum of the right lung, accompanied by extensive pleural effusion.  A smaller mass (0.5 × 1.0 × 0.7 cm) was also observed in the left lung, and an abdominal CT scan also revealed an irregular mass (1.5 × 1.0 × 0.9 cm) on the edge of the liver (Figs. 1A and B*a*, 1B*f*). Precutaneous pulmonary and disseminated pleural needle biopsies were performed, with hematoxylin and eosin (H&E) tissue staining and examination by pathologist resulting in a diagnosis of Stage IV lung squamous cell carcinoma (T4N0M1ab) with performance status (PS) of 2 (Fig. 1A).

Based on disease stage, patient age, and PS score, standard chemotherapeutic treatment consisting of repeated cycles of 5 mg/m^2^ Vinorelbine administered on Days 1 and 8 was initiated. The patient underwent nine cycles of single-agent chemotherapy at time intervals of ∼28 d over 8 mo (Fig. 1B). CT scans of the chest and abdomen performed after six cycles and nine cycles revealed significant tumor regression of both the pulmonary tumors and the liver mass (Figs. 1B*b–c*, 1B*g–h*), which was accompanied by an overall decrease in chest pain. However, the patient continued to experience frequent night sweats and fever and continued to lose weight (∼25 lb) due to the chemotherapy-related side effects of poor appetite and vomiting. Performance status was decreased to PS3 and chemotherapy had to be discontinued.

Genetic testing of a lung tumor needle biopsy specimen had revealed the presence of the common L858R driver mutation of the epidermal growth factor receptor (EGFR) gene. Second-line therapy with the EGFR inhibitor erlotinib hydrochloride (150 mg daily) was therefore initiated following cessation of maintenance chemotherapy. However, erlotinib treatment did not ease the coughing or fever symptoms, and chest pain got progressively worse. The patient also started to experience serious abdominal pain in the area near the liver metastasis. Three months following initiation of the EGFR inhibitor, CT scans of the chest and abdomen revealed a dramatic increase in the number of tumors within the right lung (Fig. 1B*d*), and significant progression of the liver tumor (measured at 4.9 × 6.7 × 5.0 cm, Fig. 1B*i*). The patient’s PS remained at 3 and erlotinib therapy was discontinued.

With erlotinib-resistant disease and low PS restricting the patient’s standard treatment options (including chemotherapy), he was offered a personalized multi-epitope peptide vaccine designed based on the unique somatic mutation profile present in his tumor, as detected with next-generation genetic sequencing. The patient expressed a strong desire to receive the vaccine and was consented accordingly. A needle biopsy of the original lung tumor was performed 2 weeks following cessation of erlotinib therapy to obtain fresh tumor tissue, which subsequently underwent mutational profiling by whole exome sequencing (WES) as well as mutational testing for a panel of 508 cancer-associated genes. Ninety-three non-synonymous somatic mutations were detected by WES, five of which were confirmed in the 508-gene test (Table 1). Mutation-containing peptide fragments encoded by the five mutated genes were assessed for predicted binding to the patient’s HLA class I and class II molecules using peptide binding prediction algorithms.^17-18^ Several mutated peptide fragments were predicted to bind to the patient’s HLA allotypes, including four peptides with very high-predicted binding affinity (Table 2). Peptides were dissolved in saline and administrated weekly in two separate peptide cocktails (five short + one long, 200 ug of each peptide), one into each arm by subcutaneous injection. Aldara cream (5% Imiquimod) was applied topically over each vaccination site immediately afterwards in order to provide immune activation co-stimulatory signals to professional antigen-presenting cells through stimulation of Toll-like receptors (TLR) 7 and 8. Other than a temporary rash occurring at the vaccine site following immunization, no adverse events were noted. No other therapy was given or received during the immunization period.

**Table 1.** Genetic sequencing analysis of patient lung tumor and HLA typing.

^1^Somatic mutations in tumor:			HLA typing results:				
Gene	DNA mutation	Predicted protein alteration	HLA-A	HLA-B	HLA-C	HLA-DQB1	HLA-DRB1
*EGFR*	2573T>G	L858R	A*1101 A*3101	B*3501 B*4006	C*0303 C*0801	DQB1*0401 DQB1*0602	DRB1*0405 DRB1*1501
*STK11*	811delA	S271A fs*16					
*NAV3*	6663C>A	F2221L					
*EPHB1*	1382A>G	N461S					
*PTCH2*	2431C>T	R811C					

1 Somatic mutations were detected by both whole exome sequencing and 508 cancer-associated gene analysis (see *Methods*).

**Table 2.** Personalized neo-epitope vaccine peptides and predicted HLA binding.

Protein	Amino acid change	^1^Mutant peptide	HLA allele	^2^Mutant peptide affinity (nM)	WT peptide affinity (nM)	Peptide designation
EGFR	L858R	HVKITDFGR	A*3101	**10**	3637	H9R9
		KITDFGRAK	A*1101	71	**18**	K9R7
		HVKITDFGRAK	A*3101	305	296	H11R9
		RAKLLGAEEK	A*3101	903	4611	R10R1
STK11	S271ins.16	ATPSRATVAPR	A*3101	53	NA	AT11*fs
		RATVAPRSL	C*0303	**20**	NA	RA9*fs
		FENIGKG*ATPSRATVAPRSLT	DQB1*0602	185	NA	FE21*fs
NAV3	F2221L	VTIGPRLLL	C*0303	**24**	**17**	V9L8
		GPRLLLPCPM	B*3501	1075	1197	G10L5
EPHB1	N461S	QPSGIILDY	B*3501	**12**	**9**	Q9S3
PTCH2	R811C	CNGSEDGALAY	B*3501	1298	4272	C11C1

1 Predicted HLA-binding mutant peptides included in the peptide vaccine. Mutated amino acid residues are underlined.

2 HLA-binding affinities of mutated and corresponding wild-type peptides, as predicted by NetMHC3.4. Bold font indicates peptides with very high-predicted HLA-binding affinity (< 50 nM).

Two weeks after initiating vaccination, the patient reported notable improvements in breathing and significant alleviation of chest pain. Cough symptoms and occurrence of fever were also reduced, and soon afterwards his PS score was raised to 2. A chest CT scan performed 38 d following the initiation of peptide vaccination showed a remarkable regression of multiple tumor nodules in the right lung compared to those observed in pre-vaccine scans (Fig. 1B*e*). By contrast, the large liver metastasis showed slight progression over the same time period (5.1 × 6.4 × 5.2 cm, Fig. 1B*j*). Despite the robust antitumor response observed in the lung, over the following 3 weeks the patient began to experience increasing abdominal pain and reduced performance status, and the peptide vaccine was discontinued after eight doses. He expired shortly afterwards from complications due to progression of the liver metastasis.

## Results

### Assessment of vaccine-induced antigen-specific cytotoxic T cell response

**Figure 2.**
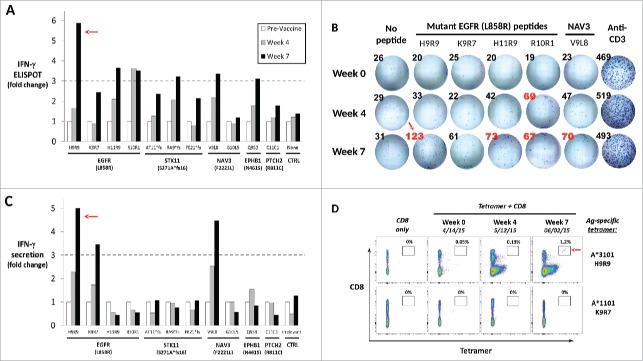
Immune monitoring of vaccination-induced peripheral blood T-cell response. Peripheral blood mononuclear cells (PBMC) collected prior to vaccination (Week 0) and at Weeks 4 and 7 post-vaccination were assessed for peptide-specific T-cell recognition using three assays. (A) Summary of IFNγ ELISPOT results showing change in peptide-specific IFNγ-producing PBMC compared with pre-vaccine levels. Dashed line indicates a 3-fold increase. (B) IFNγ ELISPOT results for the four EGFR(L858R)-derived mutant peptides compared with no peptide and anti-CD3 controls. Spot counts per well are shown, with red font indicating an increase of 3-fold or more compared to pre-vaccine levels. (C) Summary of IFNγ ELISA results showing change in peptide-specific IFNγ secretion by pre- and post-vaccine patient PBMC following 5-d culture with vaccine or control peptides. See Table 1 for peptide designation codes and full peptide sequences. (D) Flow cytometric detection of peptide antigen-specific CD8^+^ cells by specific HLA-peptide tetramer staining. Pre- and post-vaccine PBMC were stained with anti-CD8^+^ mAb *(y-axis)* alone, or in combination with HLA-peptide tetramers *(x-axis)* specific for mutated EGFR(L858R) peptides HLA-A*3101/H9R9 *(top row)* or HLA-A*1101/K9R7 *(bottom row)*. Percentages of tetramer-positive CD8^+^ T cells are indicated. All experiments were performed at least twice with comparable results, with representative experiments depicted. Red arrows indicate T-cell reactivity against the H9R9 EGFR-derived peptide, which induced the most robust T-cell responses in all three assays.

Peripheral blood was collected from the patient prior to immunization (Week 0), and again at Weeks 4 and 7 post-vaccination. To determine whether functional, antigen-specific T lymphocytes were induced by the peptide vaccine, PBMC from each time point were analyzed for interferon-γ (IFNγ) production *ex vivo* following stimulation with each of the vaccine peptides individually. As shown in Fig. 2A, IFNγ ELISPOT analysis showed that antigen-specific T-cell frequencies were significantly increased (>3-fold) against 6 of the 11 mutated peptides compared to pre-vaccine levels. Notably, three peptides eliciting the largest fold change were all derived from mutated EGFR-L858R (Fig. 2B). Pre- and post-vaccine PBMC were also analyzed for IFNγ secretion by ELISA assay following culture with individual vaccine peptides and re-stimulation on Day 7. These results corroborated the ELISPOT results for two of the three mutated EGFR-derived peptides H9R9 (HVKITDFG**R**) and K9R7 (KITDFG**R**AK), in addition to one of the NAV3-derived peptides, V9L8 (VTIGPRL**L**L, Fig. 2C).

To confirm antigen specificity, custom HLA-peptide tetramers were successfully generated for the two most reactive EGFR peptides, H9R9 (restricted to HLA*A3101) and K9R7 (restricted to HLA-A*1101). Week 0 and Week 4 PBMC demonstrated little or no specific staining with the EGFR-H9R9 tetramer. However, Week 7 post-vaccine PBMC showed a clear tetramer-positive population that constituted 1.2% of total CD8^+^ T lymphocytes (Fig. 2D). These results were consistent with the Week 7 ELISPOT results, in which ∼1% of CD8^+^ PBMC produced IFNγ in response to H9R9 peptide stimulation. By contrast, the EGFR-K9R7 tetramer did not specifically stain patient PBMC, possibly due to reduced tetramer stability resulting from the moderate predicted affinity of the K9R7 peptide for HLA-A*1101 (Table 2). In summary, immune monitoring demonstrated immune reactivity against a number of peptides, with the most robust post-vaccine CD8^+^ T-cell response detected against the HLA-A*3101-restricted EGFR-H9R9 peptide.

### Discussion

Although cancer vaccine trials have been ongoing for over two decades, objective clinical responses to vaccination have remained relatively rare, more typically resulting in modest benefits in progression-free survival.^19-22^The squamous cell carcinoma patient reported here is notable in this regard, having experienced a rapid and dramatic regression of multiple lung tumors following neo-epitope peptide vaccination. For reasons discussed below, we do not expect such a response to be typical with this vaccination approach; however, we do believe this case illuminates some critical concepts and challenges that are highly relevant to the design and potential success of future cancer vaccine trials.

One major feature that sets this approach apart from the majority of prior cancer vaccine trials is the use of patient-specific, mutated peptide antigens predicted by next-generation sequencing, which has only recently become possible to perform routinely in individual patients.^23^ Several lines of evidence support the notion that a significant proportion of antitumor T-cell responses are focused upon somatically-mutated peptides presented by either HLA class I or class II molecules.^24-25^ For example, tumor-infiltrating lymphocytes (TIL) have been shown to frequently recognize tumor-associated mutated peptide antigens; furthermore, the responses to checkpoint blockade-based immunotherapies have been well correlated with mutational burden of the tumor.^4,5,26-27^ Mutated antigens are known to be substantially more immunogenic compared with non-mutated tumor antigens, therefore significantly increasing the chances of generating a productive tumor-specific T-cell response through vaccination.^28-29^ In addition, targeting multiple, personalized peptide epitopes (in this case, 11) may have led to a broader immune response than was possible with most prior vaccine trials, which have typically focused on targeting single or just a few antigens.  Employing a saline-based vaccine rather than using Incomplete Freund’s Adjuvant (IFA) may have also led to increased vaccine efficacy, as IFA vaccine depots have been shown to attract and deplete antigen-specific T cells, which has possibly compromised the effectiveness of prior vaccine formulations.^30^

Of the 93 somatic mutations detected by WES, we chose to focus on targeting mutations from five known cancer-associated genes (EGFR, PTCH2, EPHB1, NAV3, and STK11), reasoning they would be most likely to be expressed at the protein level and least likely tolerated to be lost through immune selection and tumor escape. Mutated peptides derived from four of the five genes were predicted to bind with high affinity (Table 2). Functional immune monitoring showed that peptide vaccination induced antigen-specific CD8^+^ T cell responses by Week 7 against 6 of the 11 peptides by IFNγ ELISPOT, three of which were confirmed by ELISA. Of note, two of the three confirmed peptides (H9R9 and K9R7) were derived from the well-described shared EGFR-L858R mutation harbored by ∼20% of Asian lung cancer patients, and less frequently (∼5%) by non-Asian patients.^31^ By week 7, vaccination with the high affinity (10 nM) H9R9 peptide induced a clear population of HLA-A*3101-restricted tetramer-positive CD8^+^ cells in the patient’s peripheral blood; however, tetramer-positive PBMC with specificity for the moderate affinity (71 nM) HLA-A*1101-restricted K9R7 peptide were not detectable. Whether these predicted differences in affinity accurately reflect actual HLA-peptide binding will require further study. Though we cannot exclude the contribution of multiple peptides to the clinical response observed (for example, the STK11 frameshift mutation), the dominant T-cell response seemed to be directed toward mutated EGFR, and specifically the H9R9 peptide. Interestingly, the L858R amino acid change has the potential to create true neo-epitope through alteration of a critical anchor residue in peptide position P9, which heavily favors binding to a number of HLA-A3 family members: HLA-A*3101, A*3301, A*3303, and A*6801, collectively expressed by ∼12% of the worldwide population and ∼20% of Asians (Table 3). Thus, this tumor-associated antigen may represent a shared, neo-epitope target for a significant number of lung cancer patients worldwide, particularly those of Asian descent. In China alone, ∼40,000 new cases per year of EGFR (L858R)-positive lung cancer patients are expected to be diagnosed that express one of these HLA allotypes.^31-32^ Being derived from a driver mutation, targeting mutated EGFR-derived peptides represents a particularly promising immunotherapeutic approach since the tumor very likely cannot lose the mutated EGFR protein. However, further laboratory studies will be required to validate these L858R peptide targets as being naturally processed and presented by lung cancer cells.

**Table 3.** Mutant EGFR (L858R) peptide binding to members of the HLA-A3 superfamily.

		Predicted binding affinities of mutant peptides (nM)			HLA allotype frequencies		
Gene (mutation)	HLA allotype	HVKITDFGR	HVKITDFGRAK	KITDFGRAK	Asia	Western	Refs.
EGFR (L858R)	HLA-A03:01	11,758	4,406	221	4.2	12.4	^1^^,^^2^
	HLA-A11:01	5,607	2,680	71	28.4	6.3	^1^^,^^2^
	HLA-A30:01	837	8	48	3.8	2.1	^1^^,^^2^
	HLA-A31:01	12	305	872	5.3	10.5	^1^^,^^2^
	HLA-A33:01	46	15,553	17,651	1.8	2.4	^1^^,^^3^
	HLA-A33:03	15	6,272	5,927	10.2	1.9	^1^^,^^3^
	HLA-A68:01	13	157	3,137	2.2	5.2	^1^^,^^2^

1 http://www.allelefrequencies.net.

2 http://www.cbs.dtu.dk/services/NetMHC-3.4/.

3 http://www.cbs.dtu.dk/services/NetMHCpan/.

A number of important limitations remain to be overcome in order to further optimize the neo-epitope peptide vaccine approach. Currently, the most critical issue concerns target epitope identification: simply stated, there are substantially more (>100-fold) potential predicted mutated peptides than are actually presented by tumor cells.^33-34^ Although quantitating tumor-associated gene expression by RNAseq or measuring TIL reactivity can provide some guidance regarding epitope choice, which mutated peptides constitute bona fide tumor antigens in individual cancer patients remains largely unknown. Mass spectrometry-based methods though promising currently lack the sensitivity required to directly detect mutated peptide antigens from most patient tumor samples, which are often of limited size.^35-36^ Narrowing down the list of potential neo-epitopes is currently a major unresolved clinical challenge, particularly for highly mutated cancers such as melanoma and lung carcinoma.^6,37^

A further challenge concerns how to best increase the potency of cancer vaccines with the use of specific immune adjuvants. Several human and animal studies suggest this limitation may be overcome by employing Type I interferon-inducing TLR agonists in combination with anti-CD40 or anti-CD70, for which clinical reagents are currently available.^29^ Based on recent studies, mutated peptide-pulsed dendritic cell (DC)-based vaccines hold tremendous promise for generating effective CTL responses.^13^ Immune suppression within the tumor microenvironment also constitutes a major limitation to any immune-based therapy. However, several new agents designed to overcome this problem are currently in clinical use or in development, including T-cell checkpoint blockade approaches (anti-CTLA4, anti-PD1/PD-L1), depletion/conversion of tumor-associated myeloid cells (CSF1 inhibitors), suppressive enzyme inhibition (indolamine deoxygenase (IDO) inhibitors), or oncogene-targeted agents (BRAF-V600E inhibitors).^23^Determining the best combination of immunotherapeutic agents to use for individual patients in the context of vaccination remains a formidable clinical question.

The last critical concept illustrated by this case report relates to the highly mixed nature of the clinical response induced by the neo-epitope vaccine: Despite the fact that several tumors in the lung demonstrated a remarkable degree of regression in 6 weeks, the much larger liver metastasis remained refractory to treatment. Although it was not possible to analyze further, we speculate that this may reflect tumor heterogeneity, size of the tumor burden, the immunosuppressive microenvironment of the liver, or a combination of these factors.^38-39^ The clonal evolution of tumors suggests a potential advantage of targeting early mutations as opposed to mutations acquired late in evolution, and targeting driver mutations over passenger mutations that can more easily be lost. However, increased tumor burden contributes not only to increased tumor heterogeneity, but also to augmented tumor suppression associated with therapeutic resistance.^40^ Knowing the dominant mechanisms of immune suppression within the liver-tumor microenvironment of the patient may have enabled a rational combination therapy with which to overcome resistance. In this context, employing next-generation genetic sequencing methods such as RNAseq analysis to different tumor sites within the same patient will provide a foundation on which to build these important efforts to address tumor heterogeneity and immune suppression. The well-documented correlation of tumor burden and therapeutic resistance as illustrated here reinforces the notion that vaccinating at earlier stages of disease, or in the adjuvant setting, will likely increase the chance of positive clinical outcomes.

## Methods

### Ethics and patient consent

The personalized peptide vaccine protocol for this patient conformed to the ethical guidelines of the 1975 Declaration of Helsinki, and was approved by ethics committee of Beichen Hospital, Tianjin, China. Informed consent was obtained from the patient and his family.

### Genetic analyses

Lung tumor samples obtained by needle biopsy were analyzed using a 508 cancer-associated gene panel (BGI Diagnostics), revealing the presence of five somatic mutations (Table 1). Tumors were also analyzed by WES (GenomiCare Biotechnology Co. Ltd.), demonstrating 93 somatic mutations in total and providing confirmation of the five genetic lesions detected by the 508-gene panel. High-resolution HLA typing analysis was performed on patient peripheral blood (BGI).

### Peptides and vaccination

Mutated peptides for the personalized vaccine were chosen based on highest predicted affinity to the patient’s HLA class I and class II molecules according to the HLA-peptide prediction algorithms NetMHC3.4 (http://www.cbs.dtu.dk/services/NetMHC-3.4/) and NetMHC2.2 (http://www.cbs.dtu.dk/services/NetMHCII/). Reasoning that cancer-associated genes are more likely to be expressed at the RNA and protein levels, it was decided to design the vaccine based on mutated peptides derived from the five somatic mutations detected by the 508-gene panel. As shown in Table 1, 11 peptides (10 short and 1 long) were provided (Sangon Biotech Company Ltd. and Hang Jia Biotechnology Development Company) at >98% purity and tested for sterility and the presence of endotoxin to ensure safety and tolerability. Peptides were solubilized individually in sterile phosphate-buffered saline (PBS), mixed into two separate peptide cocktails (each with five short peptides and one long peptide in 1 mL total volume) and injected subcutaneously into the left and right extremities. The patient received 200 μg of each peptide per immunization, given weekly for 8 weeks. As a vaccine adjuvant, Aldara cream with 5% imiquimod was applied topically over the vaccine site immediately afterward.

### HLA population frequencies

Estimated HLA frequencies in Asian and Western countries in Table 3 were calculated by deriving the mean allotype frequency of the following representative nations (from http://www.allelefrequencies.net/) and normalizing for population size: Asian countries: China, Japan, Vietnam, Singapore, Thailand; Western countries: United States, England, France, Italy, Spain, Hungary, Mexico, Argentina. Worldwide HLA-A frequency estimates were derived from allotype frequencies listed at http://www.pypop.org/popdata/2008/byallele-A.php.

### Immune monitoring

Peripheral blood mononuclear cells (PBMC) were isolated by Ficoll density gradient centrifugation and counted in the presence of trypan blue dye to evaluate viability. For ELISPOT assay, 1.25 × 10^5^ PBMC per well were cultured for 36 h with individual vaccine or control peptides (5 ug/mL) in a 96-well plate and developed according to the manufacturer’s instructions (Human IFNγ ELISpot^PRO^ kit, MABTECH Inc., USA). For ELISA assay, PBMC were cultured for 5 d at 1 × 10^5^ cells per well in the presence of individual vaccine or control peptides (5 ug/mL) and 300 IU/mL interleukin(IL)-2. Cultured PBMC were re-stimulated with individual peptides on Day 5 and cell supernatants were collected 6 h later. IFNγ concentration was measured using a human IFNγ ELISA kit (Invitrogen, USA). For tetramer analysis, custom phycoerethrin(PE)-conjugated tetramers (Baylor College of Medicine, USA) were successfully generated for the H9R9 (HVKITDFG**R**) and K9R7 (KITDFG**R**AK) peptides restricted to HLA-A*3101 and HLA-A*1101, respectively. A total of 1 × 10^6^ PBMC from each time point were stained with each custom tetramer along with anti-CD8^+^-PerCP (Biolegend), and analyzed by flow cytometry.

## Supplementary Material

KONI_A_1238539_s02.pptx

## References

[1] Hecht SS. Lung carcinogenesis by tobacco smoke. Int J Cancer 2012; 131(12):2724-32; PMID:22945513; http://dx.doi.org/10.1002/ijc.27816 22945513 PMC3479369

[2] Lawrence MS, Stojanov P, Polak P, Kryukov GV, Cibulskis K, Sivachenko A, Carter SL, Stewart C, Mermel CH, Roberts SA et al. Mutational heterogeneity in cancer and the search for new cancer-associated genes. Nature 2013; 499(7457):214-8; PMID:23770567; http://dx.doi.org/10.1038/nature12213 23770567 PMC3919509

[3] Rizvi NA, Hellmann MD, Snyder A, Kvistborg P, Makarov V, Havel JJ, Lee W, Yuan J, Wong P, Ho TS et al. Cancer immunology. Mutational landscape determines sensitivity to PD-1 blockade in non-small cell lung cancer. Science 2015; 348(6230):124-8; PMID:25765070; http://dx.doi.org/10.1126/science.aaa1348 25765070 PMC4993154

[4] McGranahan N, Furness AJ, Rosenthal R, Ramskov S, Lyngaa R, Saini SK, Jamal-Hanjani M, Wilson GA, Birkbak NJ, Hiley CT et al. Clonal neoantigens elicit T cell immunoreactivity and sensitivity to immune checkpoint blockade. Science 2016; 351(6280):1463-9; PMID:26940869; http://dx.doi.org/10.1126/science.aaf1490 26940869 PMC4984254

[5] Van Allen EM, Miao D, Schilling B, Shukla SA, Blank C, Zimmer L, Sucker A, Hillen U, Geukes Foppen MH, Goldinger SM et al. Genomic correlates of response to CTLA-4 blockade in metastatic melanoma. Science 2015; 350(6257):207-11; PMID:26359337; http://dx.doi.org/10.1126/science.aad0095 26359337 PMC5054517

[6] Schumacher TN, Schreiber RD. Neoantigens in cancer immunotherapy. Science 2015; 348(6230):69-74; PMID:25838375; http://dx.doi.org/10.1126/science.aaa4971 25838375

[7] Gubin MM, Zhang X, Schuster H, Caron E, Ward JP, Noguchi T, Ivanova Y, Hundal J, Arthur CD, Krebber WJ et al. Checkpoint blockade cancer immunotherapy targets tumour-specific mutant antigens. Nature 2014; 515(7528):577-81; PMID:25428507; http://dx.doi.org/10.1038/nature13988 25428507 PMC4279952

[8] Coulie PG, Van den Eynde BJ, van der Bruggen P, Boon T. Tumour antigens recognized by T lymphocytes: at the core of cancer immunotherapy. Nat Rev Cancer 2014; 14(2):135-46; PMID:24457417; http://dx.doi.org/10.1038/nrc3670 24457417

[9] Gubin MM, Artyomov MN, Mardis ER, Schreiber RD. Tumor neoantigens: building a framework for personalized cancer immunotherapy. J Clin Invest 2015; 125(9):3413-21; PMID:26258412; http://dx.doi.org/10.1172/JCI80008 26258412 PMC4588307

[10] Robbins PF, Lu YC, El-Gamil M, Li YF, Gross C, Gartner J, Lin JC, Teer JK, Cliften P, Tycksen E et al. Mining exomic sequencing data to identify mutated antigens recognized by adoptively transferred tumor-reactive T cells. Nat Med 2013; 19(6):747-52; PMID:23644516; http://dx.doi.org/10.1038/nm.3161 23644516 PMC3757932

[11] Della Vittoria Scarpati G, Fusciello C, Perri F, Sabbatino F, Ferrone S, Carlomagno C, Pepe S. Ipilimumab in the treatment of metastatic melanoma: management of adverse events. Onco Targets Ther 2014; 7:203-9; PMID:24570590; http://dx.doi.org/10.2147/OTT.S57335 24570590 PMC3933725

[12] Haanen JB, Thienen HV, Blank CU. Toxicity patterns with immunomodulating antibodies and their combinations. Semin Oncol 2015; 42(3):423-8; PMID:25965360; http://dx.doi.org/10.1053/j.seminoncol.2015.02.011 25965360

[13] Carreno BM, Magrini V, Becker-Hapak M, Kaabinejadian S, Hundal J, Petti AA, Ly A, Lie WR, Hildebrand WH, Mardis ER et al. Cancer immunotherapy. A dendritic cell vaccine increases the breadth and diversity of melanoma neoantigen-specific T cells. Science 2015; 348(6236):803-8; PMID:25837513; http://dx.doi.org/10.1126/science.aaa3828 25837513 PMC4549796

[14] Kreiter S, Vormehr M, van de Roemer N, Diken M, Löwer M, Diekmann J, Boegel S, Schrörs B, Vascotto F, Castle JC et al. Mutant MHC class II epitopes drive therapeutic immune responses to cancer. Nature 2015; 520(7549):692-6; PMID:25901682; http://dx.doi.org/10.1038/nature14426 25901682 PMC4838069

[15] van der Burg SH, Arens R, Ossendorp F, van Hall T, Melief CJ. Vaccines for established cancer: overcoming the challenges posed by immune evasion. Nat Rev Cancer 2016; 16(4):219-33; PMID:26965076; http://dx.doi.org/10.1038/nrc.2016.16 26965076

[16] Pol J, Bloy N, Buqué A, Eggermont A, Cremer I, Sautès-Fridman C, Galon J, Tartour E, Zitvogel L, Kroemer G et al. Trial Watch: Peptide-based anticancer vaccines. Oncoimmunology 2015; 4(4):e974411; PMID:26137405; http://dx.doi.org/10.4161/2162402X.2014.974411 26137405 PMC4485775

[17] Lundegaard C, Lund O, Nielsen M. Prediction of epitopes using neural network based methods. J Immunol Methods 2011; 374(1–2):26-34; PMID:21047511; http://dx.doi.org/10.1016/j.jim.2010.10.011 21047511 PMC3134633

[18] Nielsen M, Lund O. NN-align. An artificial neural network-based alignment algorithm for MHC class II peptide binding prediction. BMC Bioinformatics 2009; 10:296; PMID:19765293; http://dx.doi.org/10.1186/1471-2105-10-296 19765293 PMC2753847

[19] Nestle FO, Alijagic S, Gilliet M, Sun Y, Grabbe S, Dummer R, Burg G, Schadendorf D. Vaccination of melanoma patients with peptide- or tumor lysate-pulsed dendritic cells. Nat Med 1998; 4(3):328-32; PMID:9500607; http://dx.doi.org/10.1038/nm0398-328 9500607

[20] Schwartzentruber DJ, Lawson DH, Richards JM, Conry RM, Miller DM, Treisman J, Gailani F, Riley L, Conlon K, Pockaj B et al. gp100 peptide vaccine and interleukin-2 in patients with advanced melanoma. N Engl J Med 2011; 364(22):2119-27; PMID:21631324; http://dx.doi.org/10.1056/NEJMoa1012863 21631324 PMC3517182

[21] Slingluff CL Jr, Petroni GR, Chianese-Bullock KA, Smolkin ME, Ross MI, Haas NB, von Mehren M, Grosh WW. Randomized multicenter trial of the effects of melanoma-associated helper peptides and cyclophosphamide on the immunogenicity of a multipeptide melanoma vaccine. J Clin Oncol 2011; 29(21):2924-32; PMID:21690475; http://dx.doi.org/10.1200/JCO.2010.33.8053 21690475 PMC3138719

[22] van Poelgeest MI, Welters MJ, van Esch EM, Stynenbosch LF, Kerpershoek G, van Persijn van Meerten EL, van den Hende M, Löwik MJ, Berends-van der Meer DM, Fathers LM et al. HPV16 synthetic long peptide (HPV16-SLP) vaccination therapy of patients with advanced or recurrent HPV16-induced gynecological carcinoma, a phase II trial. J Transl Med 2013; 11:88; PMID:23557172; http://dx.doi.org/10.1186/1479-5876-11-88 23557172 PMC3623745

[23] Bernatchez C, Cooper ZA, Wargo JA, Hwu P, Lizée G. Novel treatments in development for Melanoma. Cancer Treat Res 2016; 167:371-416; PMID:26601872; http://dx.doi.org/10.1007/978-3-319-22539-5_16 26601872

[24] Lu YC, Robbin PF. Cancer immunotherapy targeting neoantigens. Semin Immunol 2016; 28(1):22-7; PMID:26653770; http://dx.doi.org/10.1016/j.smim.2015.11.002 26653770 PMC4862880

[25] Tran E, Turcotte S, Gros A, Robbins PF, Lu YC, Dudley ME, Wunderlich JR, Somerville RP, Hogan K, Hinrichs CS et al. Cancer immunotherapy based on mutation-specific CD4+ T cells in a patient with epithelial cancer. Science 2014; 344(6184):641-5; PMID:24812403; http://dx.doi.org/10.1126/science.1251102 24812403 PMC6686185

[26] Tran E, Ahmadzadeh M, Lu YC, Gros A, Turcotte S, Robbins PF, Gartner JJ, Zheng Z, Li YF, Ray S et al. Immunogenicity of somatic mutations in human gastrointestinal cancers. Science 2015; 350(6266):1387-90; PMID:26516200; http://dx.doi.org/10.1126/science.aad1253 26516200 PMC7445892

[27] Linnemann C, van Buuren MM, Bies L, Verdegaal EM, Schotte R, Calis JJ, Behjati S, Velds A, Hilkmann H, Atmioui DE et al. High-throughput epitope discovery reveals frequent recognition of neo-antigens by CD4+ T cells in human melanoma. Nat Med 2015; 21(1):81-5; PMID:25531942; http://dx.doi.org/10.1038/nm.3773 25531942

[28] Trajanoski Z, Maccalli C, Mennonna D, Casorati G, Parmiani G, Dellabona P. Somatically mutated tumor antigens in the quest for a more efficacious patient-oriented immunotherapy of cancer. Cancer Immunol Immunother 2015; 64(1):99-104; PMID:25164877; http://dx.doi.org/10.1007/s00262-014-1599-7 25164877 PMC11028785

[29] Lizée G, Overwijk WW, Radvanyi L, Gao J, Sharma P, Hwu P Harnessing the power of the immune system to target cancer. Annu Rev Med 2013; 64:71-90; PMID:23092383; http://dx.doi.org/23455713 10.1146/annurev-med-112311-083918 23092383

[30] Hailemichael Y, Dai Z, Jaffarzad N, Ye Y, Medina MA, Huang XF, Dorta-Estremera SM, Greeley NR, Nitti G, Peng W et al. Persistent antigen at vaccination sites induces tumor-specific CD8+ T cell sequestration, dysfunction and deletion. Nat Med 2013; 19(4):465-72; PMID:23455713; http://dx.doi.org/10.1038/nm.3105 23455713 PMC3618499

[31] Tan DS, Yom SS, Tsao MS, Pass HI, Kelly K, Peled N, Yung RC, Wistuba II, Yatabe Y, Unger M et al. The international association for the study of lung cancer consensus statement on optimizing management of EGFR mutation positive non-small cell lung cancer: status in 2016. J Thorac Oncol 2016; 11(7):946-63 5 20; pii: S1556-0864(16)30458-0; PMID:27229180; http://dx.doi.org/10.1016/j.jtho.2016.05.008 27229180

[32] González-Galarza FF, Takeshita LY, Santos EJ, Kempson F, Maia MH, da Silva AL, Teles e Silva AL, Ghattaoraya GS, Alfirevic A, Jones AR et al. Allele frequency net 2015 update: new features for HLA epitopes, KIR and disease and HLA adverse drug reaction associations. Nucleic Acids Res 2015; 43:D784-8; PMID:25414323; http://dx.doi.org/22934249 10.1093/nar/gku1166 25414323 PMC4383964

[33] Kroemer G, Zitvogel L. Can the exome and the immunome converge on the design of efficient cancer vaccines? Oncoimmunology 2012; 1(5):579-580; PMID:22934249; http://dx.doi.org/10.4161/onci.20730 22934249 PMC3429561

[34] Heemskerk B, Kvistborg P, Schumacher TN. The cancer antigenome. EMBO J 2013; 32(2):194-203; PMID:23258224; http://dx.doi.org/10.1038/emboj.2012.333 23258224 PMC3553384

[35] Yadav M, Jhunjhunwala S, Phung QT, Lupardus P, Tanguay J, Bumbaca S, Franci C, Cheung TK, Fritsche J, Weinschenk T et al. Predicting immunogenic tumour mutations by combining mass spectrometry and exome sequencing. Nature 2014; 515(7528):572-6; PMID:25428506; http://dx.doi.org/10.1038/nature14001 25428506

[36] Bassani-Sternberg M, Coukos G. Mass spectrometry-based antigen discovery for cancer immunotherapy. Curr Opin Immunol 2016; 41:9-17; PMID:27155075; http://dx.doi.org/10.1016/j.coi.2016.04.005 27155075

[37] Martin SD, Brown SD, Wick DA, Nielsen JS, Kroeger DR, Twumasi-Boateng K, Holt RA, Nelson BH. Low mutation burden in ovarian cancer may limit the utility of neoantigen-targeted vaccines. PLoS One 2016; 11(5):e0155189; PMID:27192170; http://dx.doi.org/10.1371/journal.pone.0155189 27192170 PMC4871527

[38] de Bruin EC, McGranahan N, Mitter R, Salm M, Wedge DC, Yates L, Jamal-Hanjani M, Shafi S, Murugaesu N, Rowan AJ et al. Spatial and temporal diversity in genomic instability processes defines lung cancer evolution. Science 2014; 346(6206):251-6; PMID:25301630; http://dx.doi.org/10.1126/science.1253462 25301630 PMC4636050

[39] Heymann F, Tacke F. Immunology in the liver - from homeostasis to disease. Nat Rev Gastroenterol Hepatol 2016; 13(2):88-110; PMID:26758786; http://dx.doi.org/10.1038/nrgastro.2015.200 26758786

[40] Zahreddine H, Borden KL. Mechanisms and insights into drug resistance in cancer. Front Pharmacol 2013; 4:28; PMID:23504227; http://dx.doi.org/10.3389/fphar.2013.00028 23504227 PMC3596793

