# Supplementary figures and images for: Rapid tumor regression in an Asian lung cancer patient following personalized neo-epitope peptide vaccination
Source: Oncoimmunology. 2016 Oct 7;5(12):e1238539. doi: 10.1080/2162402X.2016.1238539 (PMC5214696; doi:10.1080/2162402X.2016.1238539)

## Slide 1
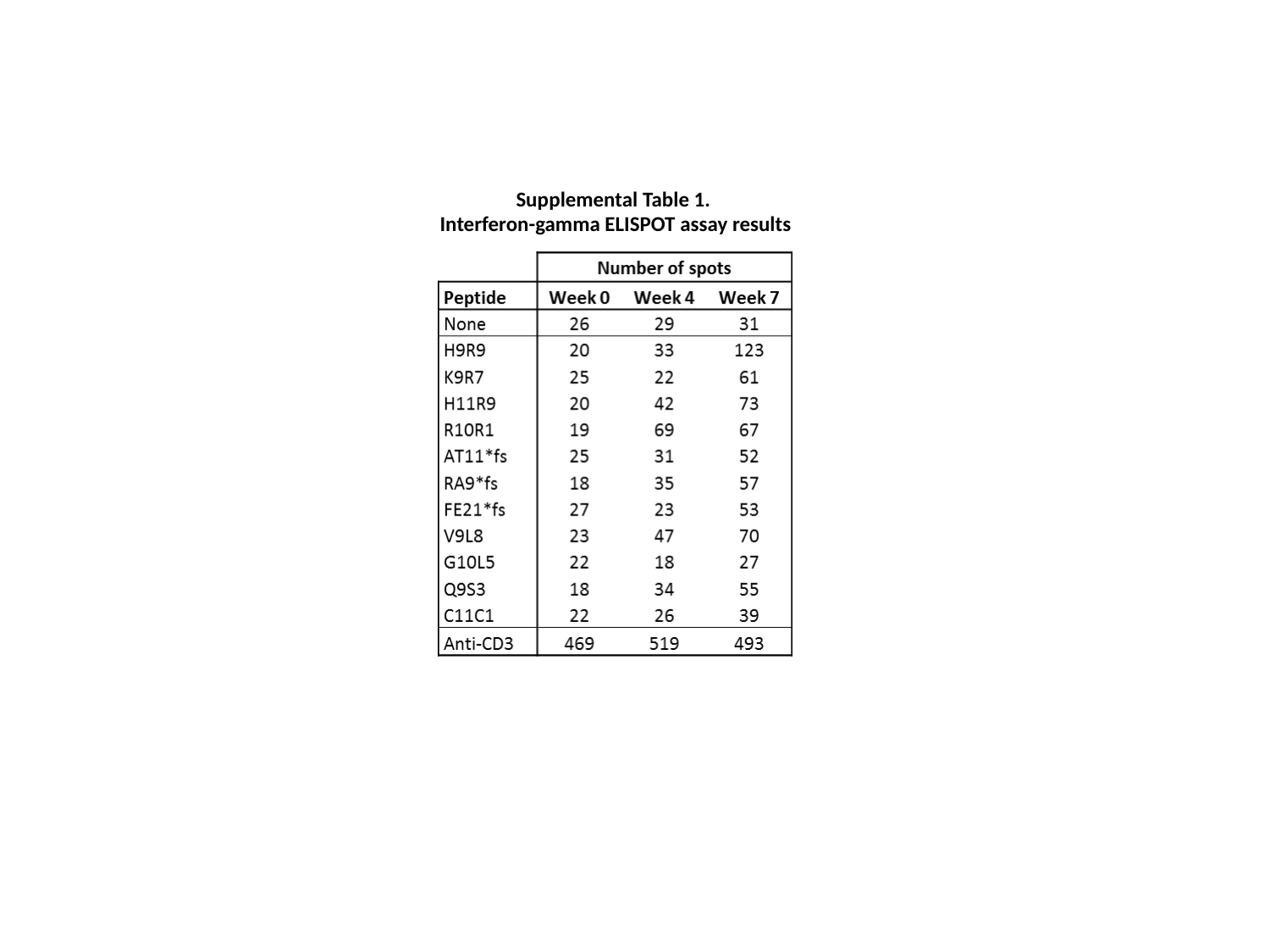

Supplemental Table 1.
Interferon-gamma ELISPOT assay results

Supplement: KONI_A_1238539_s02.pptx [file koni-05-12-1238539-s001.pptx]
